# Serological detection of *Leptospira interrogans* serovar Wolffi and clinical management of a crab-eating raccoon (*Procyon cancrivorus*) from the Brazilian Cerrado

**DOI:** 10.29374/2527-2179.bjvm002526

**Published:** 2026-08-19

**Authors:** Isabella Abreu Castro, Delcio Almeida Magalhães, Beatriz Caroline Cabral Ibelli, Laura Castro Silva, Bruna Candelori Vaz, Ana Rita Barbosa Lessa, Debora Aroeira Mueller, Márcio de Barros Bandarra

**Affiliations:** 1 Setor de Medicina de Animais Selvagens, Hospital Veterinário, Universidade Federal de Uberlândia (UFU), Uberlândia, MG, Brasil.; 2 Setor de Medicina Preventiva, Hospital Veterinário, Universidade Federal de Uberlândia (UFU), Uberlândia, MG, Brasil.; 3 Setor de Medicina de Animais Selvagens, Hospital Veterinário, Universidade Federal de Uberlândia (UFU), Uberlândia, MG, Brasil.

**Keywords:** crab-eating raccoon, leptospirosis, one health, zoonotic surveillance, guaxinim-de-mão-pelada, leptospirose, saúde única, vigilância zoonótica

## Abstract

The crab-eating raccoon (*Procyon cancrivorus*) is a widely distributed South American mesocarnivore that frequently inhabits human-modified landscapes, where it may play a role in the transmission of zoonotic pathogens. Leptospirosis is an important but underreported disease in free-ranging wildlife, particularly in South America. This report describes the clinical management of a free-ranging crab-eating raccoon rescued after a road accident in southeastern Brazil, presenting systemic, hepatic, renal, and neurological alterations. Given the epidemiological context and compatible clinical findings, serological testing for *Leptospira* spp. was performed using the microscopic agglutination test, which revealed a titer of 1:200 for serovar Wolffi. Although the titer was relatively low, it was interpreted as clinically relevant when integrated with laboratory abnormalities and clinical signs, supporting initiation of antimicrobial therapy. The animal received doxycycline and comprehensive supportive care, including analgesia, hepatoprotective treatment, nutritional support via esophageal tube, and management of neurological signs. Progressive clinical and laboratory improvement was observed, allowing tube removal after 10 days and transfer to rehabilitation. No recurrence of clinical signs was noted during a 60-day follow-up. This case highlights the importance of integrating serology, clinical evaluation, and epidemiology for the diagnosis of leptospirosis in wildlife and underscores the relevance of *P. cancrivorus* to One Health surveillance.

## Resumo

O mão-pelada (*Procyon cancrivorus*) é um mesocarnívoro sul-americano amplamente distribuído que frequentemente ocupa paisagens modificadas pelo ser humano, nas quais pode desempenhar papel na transmissão de patógenos zoonóticos. A leptospirose é uma doença importante, porém subnotificada, em animais silvestres de vida livre, especialmente na América do Sul. Este relato descreve o manejo clínico de um guaxinim-brasileiro de vida livre resgatado após atropelamento no sudeste do Brasil, que apresentava alterações sistêmicas, hepáticas, renais e neurológicas. Diante do contexto epidemiológico e dos achados clínicos compatíveis, foi realizada sorologia para *Leptospira* spp. pelo teste de soroaglutinação microscópica, que revelou título de 1:200 para o sorovar Wolffi. Embora considerado relativamente baixo, o título foi interpretado como clinicamente relevante quando integrado às alterações laboratoriais e aos sinais clínicos, sustentando a indicação de terapia antimicrobiana. O animal recebeu doxiciclina associada a cuidados de suporte abrangentes, incluindo analgesia, tratamento hepatoprotetor, suporte nutricional por sonda esofágica e manejo dos sinais neurológicos. Observou-se melhora clínica e laboratorial progressiva, permitindo a retirada da sonda após 10 dias e o encaminhamento para reabilitação. Durante 60 dias de acompanhamento, não houve recorrência de sinais clínicos. Este caso destaca a importância da integração entre sorologia, avaliação clínica e epidemiologia para o diagnóstico da leptospirose em fauna silvestre e reforça a relevância de *P. cancrivorus* no contexto de vigilância em Uma Só Saúde.

## Introduction

The crab-eating raccoon (*Procyon cancrivorus*), a medium-sized, mesocarnivorous member of the Procyonidae family, is a generalist mammal widely distributed across South American biomes, including wetlands, forests, and urban areas. Its adaptability to diverse habitats and omnivorous diet allows it to exploit a variety of food resources, contributing to its resilience in fragmented landscapes (Association of Zoos and Aquariums, 2010; Ratliff & Heatley, 2020; Teixeira & Ambrosio, 2006). Similar to the northern raccoon (*Procyon lotor*), the crab-eating raccoon interacts with humans, domestic animals, and other wildlife, and may serve as a reservoir for zoonotic pathogens (Junge et al., 2007). Understanding the ecology and health status of such generalist species is essential for assessing disease risks at the wildlife-human interface.

Leptospirosis is a globally distributed zoonosis caused by pathogenic *Leptospira* spp., affecting humans, livestock, and wildlife. Transmission typically occurs through contact with contaminated water or soil harboring leptospiral organisms excreted in the urine of infected hosts (Mapham & Vorster, 2004; Pal et al., 2021). While rodents are recognized as the primary reservoirs, numerous studies have documented leptospiral exposure in diverse wildlife species, including carnivores, marsupials, and reptiles, suggesting a broad host range and complex epidemiology (Ajayi et al., 2017; Fornazari, 2017; Fornazari et al., 2018). In Brazil, serological and molecular surveys have confirmed *Leptospira* infections in coatis (*Nasua nasua*), opossums (*Didelphis* spp.), and other free-ranging mammals, with serogroups such as Djasiman, Australis, and *L. santarosai* being prevalent. Infections have also been reported in captive crab-eating foxes (*Cerdocyon thous*), crab-eating raccoons (*P. cancrivorus*), maned wolves (*Chrysocyon brachyurus*), and giant anteaters (*Myrmecophaga tridactyla*), including the identification of serovars such as *L. interrogans* (Fornazari et al., 2018; Lilenbaum et al., 2002; Vasconcelos et al., 2021). Similarly, molecular studies in domestic dogs have revealed highly genetically conserved strains circulating across Latin America, highlighting their role in zoonotic transmission (Di Azevedo et al., 2023).

Reports of clinical leptospirosis and its management in free-ranging wildlife remain scarce, largely restricted to captive or zoo-managed animals. Notably, a Sumatran tiger (*Panthera tigris sumatrae*) with acute clinical signs of leptospirosis successfully received antimicrobial and supportive treatment, illustrating the potential for therapeutic intervention when infection is detected early (Webb et al., 2021).

Previous studies have identified *Procyon lotor* as a competent reservoir for multiple *Leptospira* serovars, including Grippotyphosa and Icterohaemorrhagiae, in urban environments (Baldi et al., 2019; Junge et al., 2007). In South America, evidence of leptospiral exposure in *P. cancrivorus* remains scarce, with only a single free-ranging individual and captive zoo-housed animals reported as positive in Brazil. In these cases, the identified agent was *Leptospira interrogans* serovar Copenhageni, belonging to the serogroup Icterohaemorrhagiae, which is commonly associated with human-impacted environments and synanthropic rodents (Fornazari et al., 2018; Lilenbaum et al., 2002; Pimentel et al., 2009). Despite limited data, these findings suggest that procyonid species play epidemiological roles in the circulation of Leptospira, highlighting their potential as links among wildlife, domestic animals, and human populations.

This report describes the clinical management of a free-ranging crab-eating raccoon that tested serologically positive for *Leptospira* serovar Wolffi at a relatively low titer while exhibiting clinically relevant signs. The case demonstrates the importance of integrating serological results with clinical evaluation to guide timely antimicrobial therapy, even when laboratory titers are not markedly elevated. The findings expand knowledge on leptospirosis in South American wildlife and provide information relevant to disease monitoring, zoonotic risk assessment, and One Health strategies.

## Case report

A free-ranging adult crab-eating raccoon (*P. cancrivorus*) was rescued from a federal highway in the Triângulo Mineiro region, in Minas Gerais, near the border with Goiás, Brazil. According to eyewitness reports, the animal had frequent contact with rural workers prior to admission.

**Figure 1:**
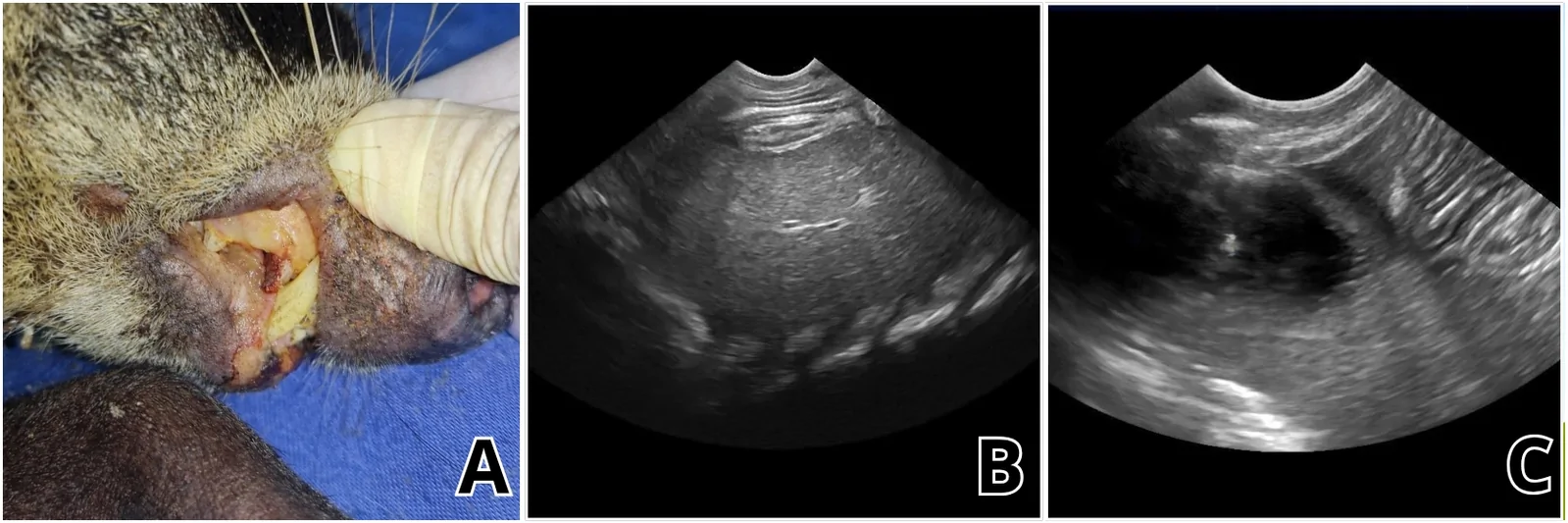
(A) Crab-eating raccoon (*Procyon cancrivorus*) presenting icteric mucous membranes at the time of admission; (B) and (C) Ultrasonographic images of the liver and gallbladder, showing a small amount of free anechoic fluid surrounding the gallbladder.

On arrival, initial stabilization followed the XABCDE trauma protocol. Physical examination revealed neurological depression, icteric mucous membranes (Figure 1A), body condition score of 6/9, and a body weight of 10 kg. Dental examination revealed complicated crown fractures with pulp exposure involving the left maxillary lateral incisor and canine tooth. Diffuse soft tissue swelling was noted in the left thoracic limb, extending from the shoulder to the manus.

Complementary diagnostics included complete blood count, serum biochemistry (alanine aminotransferase [ALT], alkaline phosphatase [ALP], albumin, total protein, urea, creatinine, triglycerides, cholesterol, calcium, and phosphorus), coagulation profile (fibrinogen, D-dimer, prothrombin time, and activated partial thromboplastin time), venous blood gas analysis, radiographic evaluation of the thoracic limb and pelvis, abdominal ultrasonography, urinalysis, and urinary protein:creatinine ratio (UPC).

Using species-specific reference intervals, hematological abnormalities included leukocytosis, neutrophilia with left shift, eosinopenia, lymphopenia, and thrombocytopenia (Table 1). Biochemical analysis revealed increased triglycerides, total protein, ALP, and ALT. Blood gas analysis showed systemic disturbances, along with hypocalcemia and hypochloremia (Table 2).

**Table 1:** Comparison between the results of hematological and its respective reference value in crab-eating racoon (*Procyon cancrivorus*).

**Parameters**	**Result**	**Reference** **1**
RBC (×10^6/uL)	7.12	7.05 ± 1.51
Hgb (g/dL)	13.1	13.77 ± 2.6
PCV (%)	40.1	39.26 ± 5.96
MCV (fL)	56.4	57.18 ± 9.56
MCH (pg)	18.3	19.96 ± 3.6
MCHC (g/dL)	32.6	34.98 ± 3.34
WBC (×10^3/uL)	17.8	9.00 ± 3.833
Bands (%)	01	-
Bands (/μL)	178	-
Segmented (%)	93	62.53 ± 14.81
Segmented (/μL)	16554	-
Eosinophils (%)	0	10.6 ± 7.3
Eosinophils (/μL)	0	-
Basophils (%)	0	-
Basophils (/μL)	0	-
Monocytes (%)	02	2.46 ± 2.09
Monocytes (/μL)	356	-
Lymphocytes (%)	04	24.40 ± 10.57
Lymphocytes (/μL)	712	-
Platelets (x 10^3^ /μL)	295	38250 ± 87567
MPV (fL)	7.5 fL	-
PDW (fL)	11.8 fL	-

1 Teixeira & Ambrosio (2006); Ratliff & Heatley (2020).

*Note.* µL = microliter; fL = femtoliters; pg = picogram; g/dL = grams per decilitre. MPV: Mean platelet volume. PDW: Platelet distribution width.

**Table 2:** Comparison between the results of biochemical and blood gas analysis and its respective reference value in crab-eating racoon (*Procyon cancrivorus*).

**Parameters**	**Result**	**Reference** **1**
Plasma protein (g/dL)	8.0	6.9 ± 0.54
Platelet Aggregation	Absent	-
Albumin (g/dL)	3.10	2.79 ± 0.4
ALT (U/L)	338	148.9 ± 104.95
Creatinine (mg/dL)	0.72	1.02 ± 0.46
ALP (U/L)	36	16.22 ± 10.88
Triglycerides (mg/dL)	69	29.86 ± 15.63
Colesterol (mg/dL)	142	113.42 ± 46
Urea (mg/dL)	18.2	41.24 ± 20.33
Sodium (mmol/L)	149.3	140 - 155^2^
Potassium (mmol/L)	4.38	3.5 - 4.5^2^
Chloride (mmol/L)	120.4	99 - 110^2^
Ionized Calcium (mmol/L)	1.21	1.3 - 1.5^2^
Glucose (mg/dL)	112	60-120^2^
Lactate (mmol/L)	1.3	0.3-2.5^2^
Anion Gap (mmol/L)	11.9	12 - 24^2^
pH (T)	7.33	7.29-7.4^3^
PCO_2_ (T) (mmHg)	40	35-41 mmHg^3^
PO_2_ (T) (mmHg)	39.3	38-44 mmHg^3^
HCO_3_ (mmol/L)	21.3	21
BE-b (mmol/L)	3.5	-4 / +4^2^

1 Teixeira & Ambrosio (2006); Ratliff & Heatley (2020);

2 Domestic dog reference;

3 Coati reference (Association of Zoos and Aquariums, 2010);

*Note.* g/dL = grams per decilitre; U/L = unit per liter; mg/dL = milligrams per deciliter; mmol/L = millimoles per liter; mmHg = millimeters of mercury.

Urinalysis revealed several abnormalities, including the presence of glucose, bile salts, mucus, occult blood, bacteria, ketones, and fat droplets in the urine. The UPC ratio was 0.74, exceeding reference values described for dogs (<0.5) and cats (<0.4), indicating clinically relevant proteinuria (Table 3). All remaining laboratory parameters were within reference limits.

**Table 3:** Comparison between the results of urinalysis and UPC and its respective reference value in crab-eating racoon (*Procyon cancrivorus*).

**Parameters**	**Result**	**Reference**
Color	Dark yellow	Yellow^1^
Aspect	Turbid	Turbid^1^
Density	1,032	1,030^1^
pH	5.5	7.5^1^
Protein	1+	1+^1^
Glucose	1+	Negative^1^
Ketone	1+	-
Occult blood	4+	-
Urobilinogen	Normal	Normal
Bilirubin	Negative	Negative^1^
Bile salts	Positive	-
Squamous epithelial cells (average per field)	0-2	-
Transitional epithelial cells (average per field)	1-4	-
Renal pelvis epithelial cells (average per field)	Absent	-
Renal tubular epithelial cells (average per field)	Absent	-
Pyocytes (average per field)	0	-
Red blood cells (average per field)	5-20	10-15
Bacteria	1+	-
Mucus	4+	-
Fat droplets	3+	-
Hyaline Cylinders	0-3	Negative
Crystals	Absent	-
UPC	0.74	<0.5^1^ or <0.4^2^

1 Domestic dog reference;

2 Domestic cat reference.

Radiographic examination revealed mild loss of serosal detail. Abdominal ultrasonography, performed in the context of polytrauma, demonstrated mild anechoic peritoneal effusion, hepatic and jejunal lymphadenomegaly with inflammatory/infectious characteristics, and splenomegaly consistent with a systemic inflammatory response. Mild dilation of the caudal vena cava and portal vein was observed, suggestive of venous congestion or stasis. Hepatic and renal architecture and echotexture were preserved, with no parenchymal lesions detected. Urinary sediment findings were compatible with an inflammatory process, suggestive of cystitis. These imaging findings were interpreted in conjunction with laboratory data to investigate hepatobiliary and renal involvement and a possible infectious etiology associated with trauma (Figures 11C).

Given the animal’s free-ranging status, epidemiological exposure, and previous reports of leptospirosis in phylogenetically related species such as northern raccoon (*Procyon lotor*), serological screening for *Leptospira* spp. was performed. The microscopic agglutination test (MAT) was selected as the serological gold standard for clinical diagnosis due to its established sensitivity, specificity, and relative accessibility for routine use. Although molecular diagnostics such as PCR offer high specificity, urine, considered the preferred specimen for detection, carries an increased risk of false-negative results depending on the stage of infection. In addition, logistical and financial constraints at the time of evaluation further supported MAT over PCR, allowing for timely and clinically actionable results.

The following serogroups and serovars were tested: Australis (*L. interrogans* serovar Bratislava), Ballum (*L. borgpetersenii* serovar Castellonis), Canicola (*L. interrogans* serovar Canicola), Djasiman (*L. interrogans* serovar Djasiman), Grippotyphosa (*L. kirschneri* serovar Grippotyphosa), Hebdomadis (*L. interrogans* serovar Hebdomadis), Icterohaemorrhagiae (*L. interrogans* serovars Copenhageni and Icterohaemorrhagiae), Pomona (*L. interrogans* serovar Pomona), Sejroe (*L. interrogans* serovars Hardjoprajitno and Wolffi), and Tarassovi (*L. borgpetersenii* serovar Tarassovi). Titers ≥1:100 with at least 50% agglutination were considered positive, following the cutoff routinely adopted by the diagnostic facility for leptospirosis serology (Gomes et al., 2023).

The animal tested positive with an antibody titer of 1:200 for serovar Wolffi, previously reported in humans, dogs, horses, and domestic ruminants. While this relatively low titer could reflect either prior exposure or early-stage infection, its interpretation was supported by the presence of clinically relevant signs. Given the absence of species-specific reference standards, the titer was considered sufficient to justify antimicrobial therapy.

Supportive therapy included analgesia with dipyrone/metamizole at 25 mg/kg q12h and tramadol hydrochloride at 3 mg/kg q12h, along with fluid therapy using lactated Ringer’s solution at 4 mL/kg/h for three days. Specific antimicrobial treatment for leptospirosis consisted of doxycycline at 5 mg/kg q12h for 14 days. Hepatoprotective therapy included silybin (*Silybum marianum*) at 8 mg/kg q12h, S-adenosylmethionine (SAMe) at 20 mg/kg q24h, vitamin E at 10 UI/kg q24h, and ursodeoxycholic acid at 10 mg/kg q12h, all administered for 21 days.

Although the patient initially showed clinical improvement, it subsequently developed head pressing, severe pain during drinking and feeding, adipsia, and anorexia. Neurological evaluation raised suspicion of central encephalic involvement, and prednisolone at 1 mg/kg q24h for 14 days was instituted. The animal was referred for surgical extraction of the fractured teeth and placement of an esophageal feeding tube using a 16 G gastric tube. Nutritional support was provided according to the patient's energy requirements using a hypercaloric moist diet (Recovery, Royal Canin, Descalvado, São Paulo, Brazil).

Progressive clinical and laboratory improvement was observed during daily evaluations. The animal gradually resumed voluntary intake of solid food, receiving a varied diet consisting of commercial dog food, fruits, vegetables, leaves, and animal protein sources, including mice (*Mus musculus*), chicks (*Gallus domesticus*), superworm larvae (*Tenebrio molitor*), and chicken eggs, in accordance with established husbandry guidelines for procyonids.

Marked improvement was noted in mastication, fine motor skills of the forelimbs, mucous membrane coloration, and laboratory parameters. The esophageal tube was removed after 10 days, reflecting the animal’s satisfactory recovery.

At the conclusion of treatment, all laboratory tests were repeated (Table 2), showing substantial improvement. Paired serological testing was also performed and yielded negative results. The animal was transferred to an external rehabilitation enclosure, and during a 60-day follow-up period, no clinical signs related to leptospirosis were observed.

## Discussion

Leptospirosis is an important consideration in the differential diagnosis of free-ranging wildlife presenting with systemic illness, particularly in tropical countries such as Brazil, where favorable environmental conditions and land-use changes facilitate pathogen circulation (Browne et al., 2022; Fornazari et al., 2018). In wild mammals, clinical manifestations are frequently nonspecific and may overlap with trauma-related inflammatory responses, hepatopathies, nephropathies, or other infectious diseases, complicating clinical recognition and delaying targeted therapy.

Serological investigation using the microscopic agglutination test (MAT) remains the most widely applied diagnostic tool for leptospirosis in animals, including wildlife. However, interpreting MAT results is challenging due to cross-reactivity, the lack of species-specific cutoffs, and the difficulty of distinguishing prior exposure from active infection (Oliveira et al., 2017). In wildlife species, these limitations are amplified by the lack of validated reference standards, reinforcing the need to interpret titers alongside clinical and epidemiological findings.

In the present case, the MAT titer of 1:200 for serovar Wolffi could be considered relatively low. Nevertheless, low titers do not preclude clinically relevant infection, particularly in early disease stages. Rapid changes in antibody titers have been documented in nondomestic species, such as a Sumatran tiger that demonstrated a sharp increase from 1:400 to 1:3,200 within 48 hours while already exhibiting clinical leptospirosis (Webb et al., 2021). This dynamic serological behavior supports clinical decision-making based on compatible signs rather than reliance on a single cutoff value.

Members of the genus *Procyon* are recognized hosts of *Leptospira* spp., especially *Procyon lotor*, which has been extensively studied as a reservoir species. Serological surveys have demonstrated exposure to multiple pathogenic serovars in urban and periurban raccoon populations (Baldi et al., 2019; Junge et al., 2007). Histopathological studies have further documented interstitial nephritis associated with leptospiral infection, supporting renal involvement even in the absence of overt clinical disease (Hamir et al., 2001).

Although renal disease is the most frequently described manifestation in *Procyon* spp., neurological involvement should not be overlooked. *Procyon lotor* is susceptible to various inflammatory and infectious encephalopathies, and secondary encephalitis has been documented in this genus (Hamir, 2011). In this context, the neurological deterioration observed in the present case may plausibly be associated with leptospiral infection, particularly given the concomitant systemic inflammatory and hepatic abnormalities, although definitive confirmation was not possible.

The identification of serovar Wolffi in a free-ranging crab-eating raccoon is noteworthy, as this serovar is more commonly associated with livestock, particularly cattle, in Brazil (Furquim et al., 2021). Serovar Wolffi has also been reported in equines with extensive geographic movement, suggesting circulation at the livestock-environment interface and potential for cross-species transmission (Vasconcelos, 2021).

In the present case, the infection was specifically attributed to *Leptospira interrogans* serovar Wolffi, which must be clearly distinguished from the bacterial species *Leptospira wolffii*. Although these terms are frequently confused in the literature, they represent distinct taxonomic entities with different epidemiological and clinical implications. While *L. wolffii* was initially regarded as a species of uncertain pathogenicity, subsequent phylogenetic and clinical evidence has demonstrated its inclusion within pathogenic or intermediate clades and its association with severe disease in humans and animals, including hepatic, renal, respiratory, and neurological involvement (Tasnim et al., 2025; Zakeri et al., 2010). This clarification reinforces the clinical relevance of infections caused by serovar *Wolffi* and highlights the unexpected nature of its detection in a free-ranging wild animal.

The identification of serovar Wolffi in wildlife is likely associated with anthropogenic environmental disturbances, particularly habitat fragmentation, deforestation, and the replacement of native ecosystems by agricultural landscapes. These processes increase overlap among wildlife, domestic animals, and human-modified environments, facilitating the transmission and persistence of leptospires typically associated with peri-urban or rural settings (Zakeri et al., 2010).

From a One Health perspective, the detection of pathogenic *Leptospira* in a free-ranging wild carnivore reflects the consequences of environmental disturbance, agricultural expansion, and increased contact among wildlife, domestic animals, and humans. Wildlife may act as sentinels of environmental contamination, signaling risks to animal production systems and public health (Sohn-Hausner et al., 2023; Sykes et al., 2022). Integrated surveillance across human, animal, and environmental sectors is therefore essential for understanding and mitigating leptospirosis transmission.

## Conclusions

This case highlights leptospirosis as a clinically relevant differential diagnosis in free-ranging *P. cancrivorus* presenting with systemic and neurological signs in endemic regions. Relatively low MAT titers should be interpreted with caution and in association with clinical and epidemiological evidence, particularly in wildlife species lacking validated serological reference values. The detection of serovar Wolffi reinforces the importance of wildlife-livestock-environment interfaces in leptospiral transmission and underscores the value of a One Health approach to disease surveillance and management.
